# [^99m^Tc]Tc-Galacto-RGD_2_ integrin α_v_β_3_-targeted imaging as a surrogate for molecular phenotyping in lung cancer: real-world data

**DOI:** 10.1186/s13550-021-00801-x

**Published:** 2021-06-13

**Authors:** Jingjing Fu, Yan Xie, Tong Fu, Fan Qiu, Fei Yu, Wei Qu, Xiaochen Yao, Aiping Zhang, Zhenhua Yang, Guoqiang Shao, Qingle Meng, Xiumin Shi, Yue Huang, Wei Gu, Feng Wang

**Affiliations:** 1 Department of Nuclear Medicine, Nanjing First Hospital, Nanjing Medical University, 68 Changle Road, Nanjing, 210006 China; 2 Department of Imaging, Nanjing First Hospital, Nanjing Medical University, Nanjing, 210006 China; 3 Department of Thoracic Surgery, Nanjing First Hospital, Nanjing Medical University, Nanjing, 210006 China; 4 Department of Respiratory, Nanjing First Hospital, Nanjing Medical University, 68 Changle Road, Nanjing, 210006 China; 5 Department of Pathology, Nanjing First Hospital, Nanjing Medical University, 68 Changle Road, Nanjing, 210006 China

**Keywords:** Lung cancer, Angiogenesis, Integrin α_v_β_3_, [^99m^Tc]Tc-Galacto-RGD_2_, Biological behaviour

## Abstract

**Background:**

Epidermal growth factor receptor tyrosine kinase inhibitors (TKIs) are beneficial in patients with lung cancer. We explored the clinical value of [^99m^Tc]Tc-Galacto-RGD_2_ single-photon emission computed tomography (SPECT/CT) in patients with lung cancer, integrin α_v_β_3_ expression, and neovascularization in lung cancer subtypes was also addressed.

**Methods:**

A total of 185 patients with lung cancer and 25 patients with benign lung diseases were enrolled in this prospective study from January 2013 to December 2016. All patients underwent [^99m^Tc]Tc-Galacto-RGD_2_ imaging. The region of interest was drawn around each primary lesion, and tumour uptake of [^99m^Tc]Tc-Galacto-RGD_2_ was expressed as the tumour/normal tissue ratio(T/N). The diagnostic efficacy was evaluated by receiver operating characteristic curve analysis. Tumour specimens were obtained from 66 patients with malignant diseases and 7 with benign disease. Tumour expression levels of α_v_β_3_, CD31, Ki-67, and CXCR4 were further analysed for the evaluation of biological behaviours.

**Results:**

The lung cancer patients included 22 cases of small cell lung cancer (SCLC), 48 squamous cell carcinoma (LSC), 97 adenocarcinoma (LAC), and 18 other types of lung cancer. The sensitivity, specificity, and accuracy of [^99m^Tc]Tc-Galacto-RGD_2_ SPECT/CT using a cut-off value of T/N ratio at 2.5 were 91.89%, 48.0%, and 86.67%, respectively. Integrin α_v_β_3_ expression was higher in non-SCLC compared with SCLC, while LSC showed denser neovascularization and higher integrin α_v_β_3_ expression. Integrin α_v_β_3_ expression levels were significantly higher in advanced (III, IV) than early stages (I, II). However, there was no significant correlation between tumour uptake and α_v_β_3_ expression.

**Conclusions:**

[^99m^Tc]Tc-Galacto-RGD_2_ SPECT/CT has high sensitivity but limited specificity for detecting primary lung cancer, integrin expression in the tumour vessel and tumour cell membrane contributes to the tumour uptake.

## Background

Lung cancer is the leading cause of cancer mortality worldwide [1, 2]. The incidence and mortality of lung cancer in China have increased rapidly in the last three decades, associated with increases in air pollution and tobacco consumption [3, 4]. However, new clinical treatment strategies, such as antiangiogenic epidermal growth factor receptor-tyrosine kinase inhibitors (EGFR-TKIs) and immunotherapy, have significantly improved the outcomes of patients with lung cancer in the last decade [5]. TKIs have a cytostatic effect on tumour cells by slowing their growth and preventing the development of distant metastases [6, 7]. Multiplex genetic sequencing has been used to select appropriate treatment, based on the recommendation of the American Society of Clinical Oncology (ASCO); however, this requires obtaining enough tumour tissue by biopsy or surgery. Unfortunately, suitable tumour specimens are unavailable for some patients due to the tumour heterogeneity or undetermined primary lesion.

Angiogenesis plays important roles in tumour initiation, development, and metastasis [8]. Integrins are a diverse family of glycoproteins that form heterodimeric receptors for extracellular matrix molecules [9–11], of which integrin α_v_β_3_, with an exposed arginine-glycine-aspartate (RGD) tripeptide sequence, is the most-extensively studied [11]. Integrin α_v_β_3_ is highly expressed in the neovasculature in solid tumours, including neuroblastoma, osteosarcoma, glioblastoma, breast cancer, and prostate cancer [12–20]. The highly restricted expression of integrin α_v_β_3_ in normal tissues compared with its overexpression in tumour cells suggests that it may provide an interesting molecular target for the early detection of malignant tumours [12]. Overexpression of integrin α_v_β_3_ was also correlated with tumour invasiveness in breast cancer, indicating a possible role in evaluating metastatic potential [19].

Radiolabelled RGD peptide as a target ligand for angiogenesis imaging has been well documented in preclinical and clinical studies [12, 21, 22]. In a previous multicentre study, we showed that [^99m^Tc]Tc labelled RGD dimers, such as [^99m^Tc]Tc-3PRGD_2_, had high sensitivity for the detection of lung cancer, including primary and metastatic tumours [21, 23, 24]**. [**^99m^Tc]Tc-Galacto-RGD_2_, with higher affinity to α_v_β_3_ and a favourable biodistribution, has been synthesized and utilized for the quantitative evaluation of α_v_β_3_ expression and of tumour angiogenesis [25].

Clinically, multiple lymphadenopathy and remote metastasis were developed rapidly in higher aggressive lung cancer even with radical resection and comprehensive treatment, we suppose some key molecules medicate the tumour development and metastasis. Therefore, we conducted a longitudinal study to evaluate the clinical role of [^99m^Tc]Tc-Galacto-RGD_2_ SPECT/CT in a large population of patients with lung neoplasms. We also explored the expression of integrin α_v_β_3_ protein in tumour cells and in the neovasculature, and determined the capability of the technique to detect lymphadenopathy and bone metastasis in patients with advanced lung cancer. Herein, we investigated the value of RGD-based imaging as a surrogate for molecular phenotyping in lung cancer.

## Methods

### Patients

**Fig. 1:**
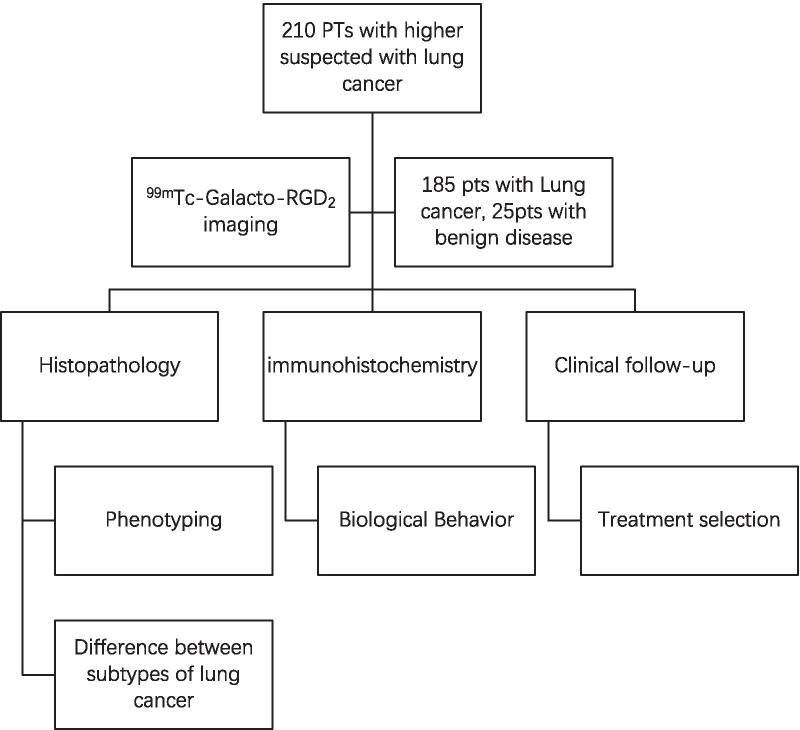
The study flow for the [^99m^Tc]Tc-Galacto-RGD_2_ imaging in the diagnosis of lung cancer

This prospective, single-centre study enrolled patients referred to our centre with suspected lung neoplasms from January 2013 to December 2016. [^99m^Tc]Tc-Galacto-RGD_2_ SPECT/CT was performed in all patients; the final diagnosis was confirmed by histopathology based on acupuncture biopsy or surgery. A total of 210 consecutive patients (147 male, 63 female; mean age 63.80 ± 10.51 years, range 21–85 years) were enrolled and analysed. Of the 210 patients, 185 were confirmed with lung cancer and the other 25 patients had benign pulmonary diseases and served as the control. Patients who had undergone perioperative chemotherapy or radiotherapy were excluded from this study; the schema of study is shown in Fig. 1.

### [^99m^Tc]Tc-Galacto-RGD_2_ radiolabelling and quality control

[^99m^Tc]Tc-Galacto-RGD_2_ labelling was carried out as described previously [25]. The Galacto-RGD_2_ was friendly offered by the School of Health Sciences, Purdue University (Indiana, USA). Chemicals were purchased from Sigma-Aldrich (St. Louis, MO). Na[^99m^Tc]TcO_4_ was obtained from DongCheng Pharmaceutical Company (Nanjing, China). Briefly, radiolabelling with performed with a lyophilized kit formulation containing 20 μg, 7 mg TPPTS (trisodium triphenylphosphine-3,3′,3″-trisulfonate), 6.5 mg tricine, 40 mg mannitol, 38.5 mg disodium succinate hexahydrate, and 12.7 mg succinic acid. ^99m^Tc-labelling was accomplished by adding 1–1.5 mL of Na[^99m^Tc]TcO_4_ solution (1,110–1,850 MBq). The reconstituted vial was heated at 100 °C for 30 min and the resulting solution was analysed by radio-high-performance liquid chromatography using a Lab Alliance system equipped with a ram IN-US detector and Zorbax C18 column (4.6 mm × 250 mm, 300 Å pore size, Waters Xbridge C18, Milford, MA). The flow rate was 1 mL/min, the mobile phase was isocratic with 90% solvent A (25 mM NH_4_OAc buffer, pH 6.8) and 10% solvent B (acetonitrile) at 0–5 min, followed by a gradient mobile phase from 10% B at 5 min to 40% B at 20 min. The radiochemical purity was > 95% for all imaging.

### [^99m^Tc]Tc-Galacto-RGD_2_ imaging and interpretation

The radiochemical purity was 95.1% ± 2.9%. [^99m^Tc]Tc-Galacto-RGD_2_ was administered at 555–740 MBq (15–20 mCi) and whole-body images were acquired at 1-h post-injection. The chest images, including the upper abdomen and adrenal glands, were performed using a combined transmission and emission device with x-ray tube and detector. All-purpose collimator centred on the140-keV energy peak with a 20% symmetrical energy window. Thirty projection images were acquired over a 180° arc at 6° intervals for each SPECT head. The acquisition time was 30 s at each projection. The transaxial data were reconstructed using Ordered Subset Expectation Maximization, 2 iterations, 8 subsets (Symbia T6 SPECT/CT; Siemens AG, Germany). Anatomic CT images were performed for attenuation correction and tumour localization. If unexpected lesions were detected by whole-body imaging, additional abdomen or pelvis images were also acquired.

All images were interpreted independently on the computer monitor in three orthogonal planes by nuclear medicine physicians and a radiologist who were unaware of the clinical information and other imaging examinations. Significantly greater local uptake of [^99m^Tc]Tc-Galacto-RGD_2_ compared with the adjacent surrounding lung was interpreted as demonstrating a malignant lesion, and uptake less than or equal to the adjacent or surrounding lung was interpreted as a benign lesion. Focal activity in the hilum and mediastinum greater than the surrounding mediastinal activity was interpreted as lymphadenopathy. Regions of interest **(**ROI) were drawn around the primary lesion and contralateral lung tissue, respectively, and [^99m^Tc]Tc-Galacto-RGD_2_ uptake was measured and expressed as the tumour/normal tissue ratio (T/N).

### Composite reference standard

All available cytologic, histologic, follow-up, and imaging findings were used as a composite reference standard for the presence of tumour lesions. This is considered the optimal gold standard because cytologic or histologic verification of every lesion was not feasible or justifiable in these patients. Whenever possible, new findings on [^99m^Tc]Tc-Galacto-RGD_2_ SPECT-CT were verified by additional investigations.

### Immunohistochemistry (IHC) analysis

Tumour specimens were obtained from patients who underwent complete resection or biopsy. The sections were fixed in formalin, embedded in paraffin, deparaffinized, and stained with haematoxylin and eosin (H&E). Integrin α_v_β_3_, Ki-67, CXCR4, and CD31 expression were analysed by IHC to evaluate the biological tumour behaviour. Sections were cut at 3-μm, dewaxed in xylene, and rehydrated in graded ethanols. Integrin α_v_β_3_ and CXCR4 expression, microvessel density (CD31), and tumour cell proliferation (Ki-67) were detected by incubating the slides with monoclonal antibodies against human integrin α_v_β_3_ (1:200, sc-7312; Santa Cruz Biotechnology, Santa Cruz, California, US), CXCR4 (1:100, ab227767; Abcam, Massachusetts, US), Ki-67 (1:100, ab270650; Abcam), or CD31 (1:50, ab28364; Abcam), respectively, overnight, followed by horseradish peroxidase-conjugated anti-mouse IgG (1:1000, Earth Ox, Millbrae, California, US) with 3′3-diaminobenzidine as the chromogen. H&E staining was also performed. All images were obtained at 100 × magnification with the same exposure time. Brightness and contrast were adjusted similarly in all images. Integrin α_v_β_3_ and CXCR4 expression levels were quantified by determining the optical density (OD) after immunostaining.

### Statistical analysis

All statistical analyses were carried out using R software (version 3.6.1) and graphs were constructed using GraphPad Prism software (version 7). Continuous variables with a non-normal distribution were expressed as median (interquartile range). Differences in T/NT and protein expression levels among groups were compared using Wilcoxon’s rank-sum or Kruskal–Wallis tests. The sensitivity, specificity, area under the curve (AUC), and cut-off value of T/NT were evaluated by receiver operating characteristic curve (ROC) analysis. Correlations between continuous variables with non-normal distributions were evaluated by Spearman’s rank correlation analysis. Bonferroni’s correction was applied for multiple comparisons. Statistical significance was established at *p* < 0.05.

## Results

### Patient characteristics

The clinical characteristics of the patients are shown in Table 1. Of the 210 consecutive patients enrolled in this study, 185 (88.1%) had malignant neoplasms identified by histopathology, including 22 patients with small cell lung cancer (SCLC), 97 with adenocarcinoma (LAC), 48 with squamous cell carcinoma (LSC), and 18 patients with other malignant lung tumours. Tumour tissues were obtained during thoracic surgery (*n* = 118), fine-needle aspiration (*n* = 35), or bronchoscopy (*n* = 32). Of the 25 patients with benign respiratory diseases, the benign nature of the lesion was confirmed during clinical follow-up in 12 patients, by histopathology in 7 patients, and at imaging follow-up in 6 patients. According to the Tumour, Node, and Metastasis **(**TNM) classification of lung cancer 8^th^ edition published in 2015 [26], 37 patients were diagnosed with stage I (20.00%), 13 with stage II (7.03%), 40 with stage III (21.62%), and 95 patients with stage IV (51.35%). The volume of the primary tumour (median (interquartile range): 28.01 (12.30, 76.33) mm^3^ was significantly higher in patients with malignant compared with benign disease (10.89 (8.66, 15.77) mm^3^) **(**Wilcoxon’s rank-sum test, *p* < 0.01).

**Table 1:** Clinical characteristics of 210 subjects

Variants	Lung cancer	Benign disease	*p*
*General*			
Age (years)	64.17 ± 10.15	61.04 ± 12.77	0.25
Sex			
Male	133 (56.00%)	14 (71.89%)	
Female	52 (44.00%)	11 (28.11%)	0.16
*Cancer type*			
LAC	97 (52.43%)		
LSC	48 (25.95%)		
SCLC	22 (11.89%)		
Other	18 (9.73%)		
*Stage*			
I	37 (20.00%)		
II	13 (7.03%)		
III	40 (21.62%)		
IV	95 (51.35%)		

*LAC* adenocarcinoma, *LSC* squamous cell carcinoma, *SCLC* small cell lung cancer

### [^99m^Tc]Tc-Galacto-RGD_2_ imaging and interpretation

**Fig. 2:**
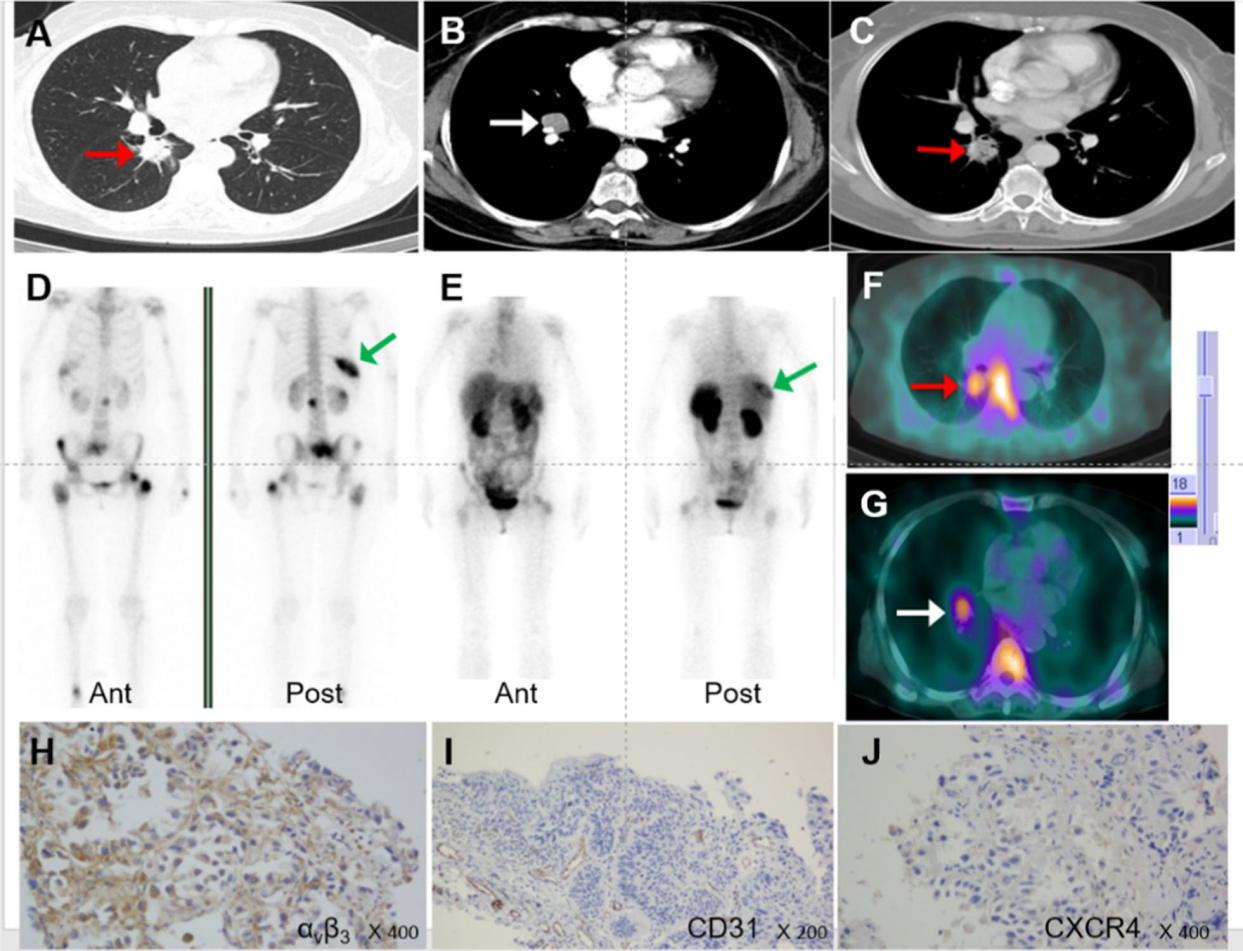
[^99m^Tc]Tc-Galacto-RGD_2_ imaging showed RGD-avid uptake in the primary tumour, lymphadenopathy, and remote metastases in a patient with suspected multiple myeloma. The final diagnosis was lung adenocarcinoma confirmed by bronchoscopic biopsy. **a** Primary lesion presented in the lung window. **b**, **c** Enhanced CT showed primary tumour and lymphadenopathy in the right hilum. **d**, **e** Bone scan showed lytic lesion in the right rib and sclerotic lesions in the pelvis. **f**, **g** ^99m^Tc-Galacto-RGD_2_ image showed avid lesions in the right lung and the right hilum. **h**, **i**, **j** IHC staining. **h** α_v_β_3_ expression. **i** CD31 expression in neo-vasculature. **j** CXCR4 expression in tumour tissue

**Fig. 3:**
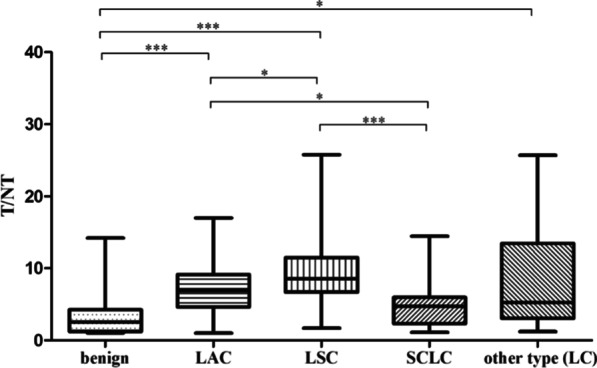
Ratio of primary tumour to normal tissue (T/NT) in different groups. *p < 0.05; **p < 0.01; ***p < 0.001

**Fig. 4:**
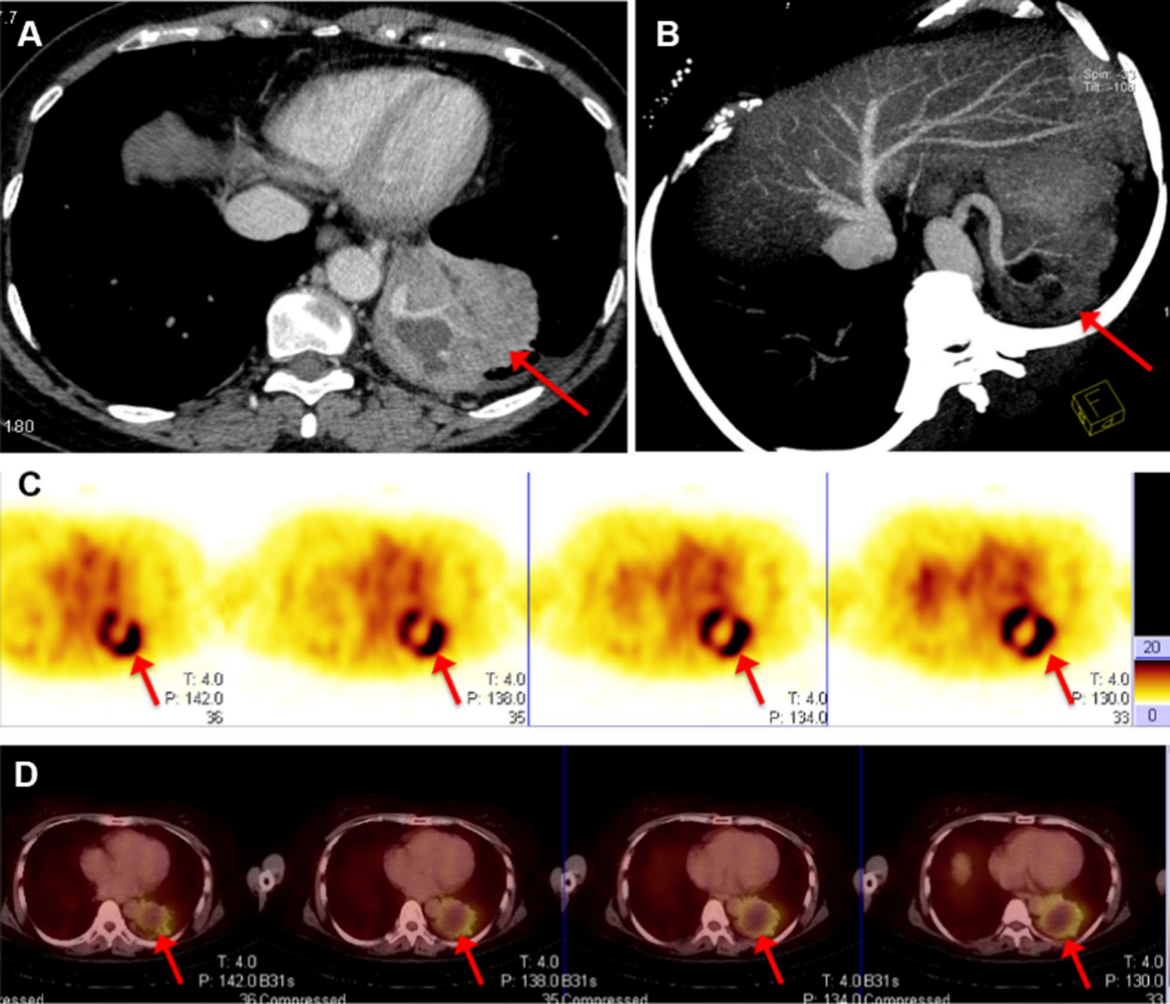
[^99m^Tc]Tc-Galacto-RGD_2_ imaging showed focal uptake in a patient with pulmonary sequestration. **a**, **b** Enhanced CT showed the tumour blood supply originated from the abdominal aorta. **c**, **d** RGD imaging showed focal uptake in the tissue, and necrosis in the central zone of the tissue

**Fig. 5:**
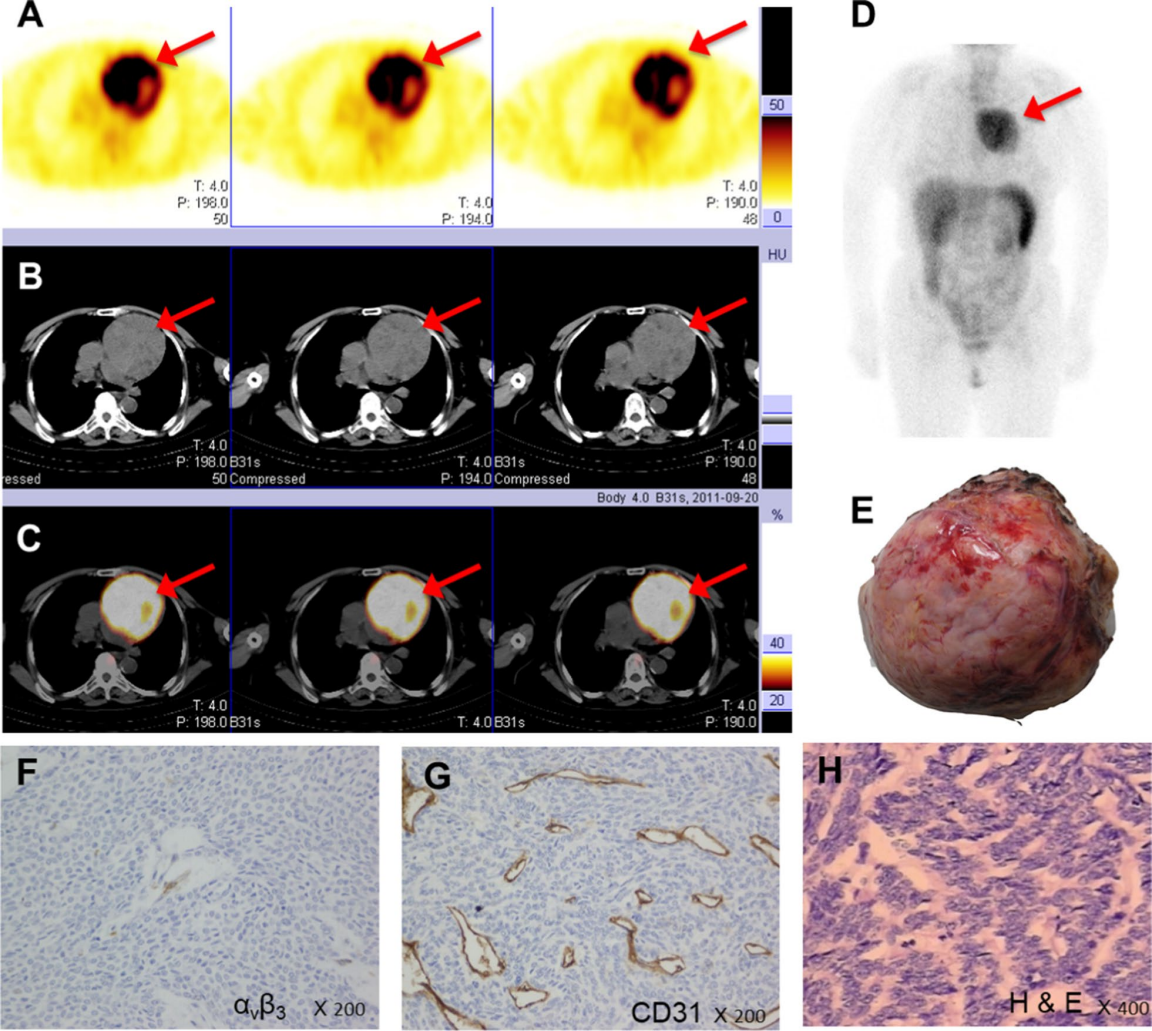
Female patient with an anterior mediastinum lesion, the final diagnosis was thymus adenoma. **a**–**d** [^99m^Tc]Tc-Galacto-RGD2 imaging showed high focal uptake. **e** The surface of the tumour showed many new vessels. **f**, **g** IHC showed a high density of neovascularization with high expression of CD31, but no significant α_v_β_3_ expression in the tumour cell membrane. **h** H&E staining

High-contrast images acquired 1 h after injection of [^99m^Tc]Tc-Galacto-RGD_2_ showed higher focal uptake in malignant primary tumours and metastatic lymph nodes (Fig. 2), compared with significantly lower uptake in benign lesions, the ratio (median (interquartile range)) of T/N in malignant disease was 6.84 (4.62, 9.86), whereas that of benign diseases was 2.53 (1.24, 3.91), *p* < 0.01. We also compared the uptake in different lung cancer subtypes (Fig. 3). [^99m^Tc]Tc-Galacto-RGD_2_ uptake was highest in LSC (T/N: 8.53 (6.75, 10.99)), followed by LAC (T/N: 6.84 (4.64, 9.07)) and SCLC (T/NT: 4.73 (2.47, 5.85)). Other types of lung cancer (T/NT: 5.23 (3.32, 11.50)) showed moderate uptake of [^99m^Tc]Tc-Galacto-RGD_2_ in the primary tumour, with no significant difference between other types and LSC, LAC, and SCLC. We also compared uptake by the primary tumour between locoregional and advanced stages. T/N was significantly lower in stage I–II (5.78 (3.62, 7.95)) compared with advanced stages (III –IV; 7.28 (5.43, 10.34)), *p* < 0.01. However, there was overlap with inflammatory pseudotumours or tuberculosis. RGD avidity was found in in two cases of pulmonary sequestration and thymoma, respectively, due to higher density of micro-vessels (Figs. 4, 5). ROC analysis indicated that the sensitivity, specificity, and accuracy of [^99m^Tc]Tc-Galacto-RGD_2_ were 91.89%, 48.0%, and 86.67%, respectively, using a cut-off value of 2.5. With a T/N cut-off value of 3.94, the AUC was 0.83 and the sensitivity and the specificity were 82.7% and 76.0%, respectively.

### Histopathology and IHC

**Fig. 6:**
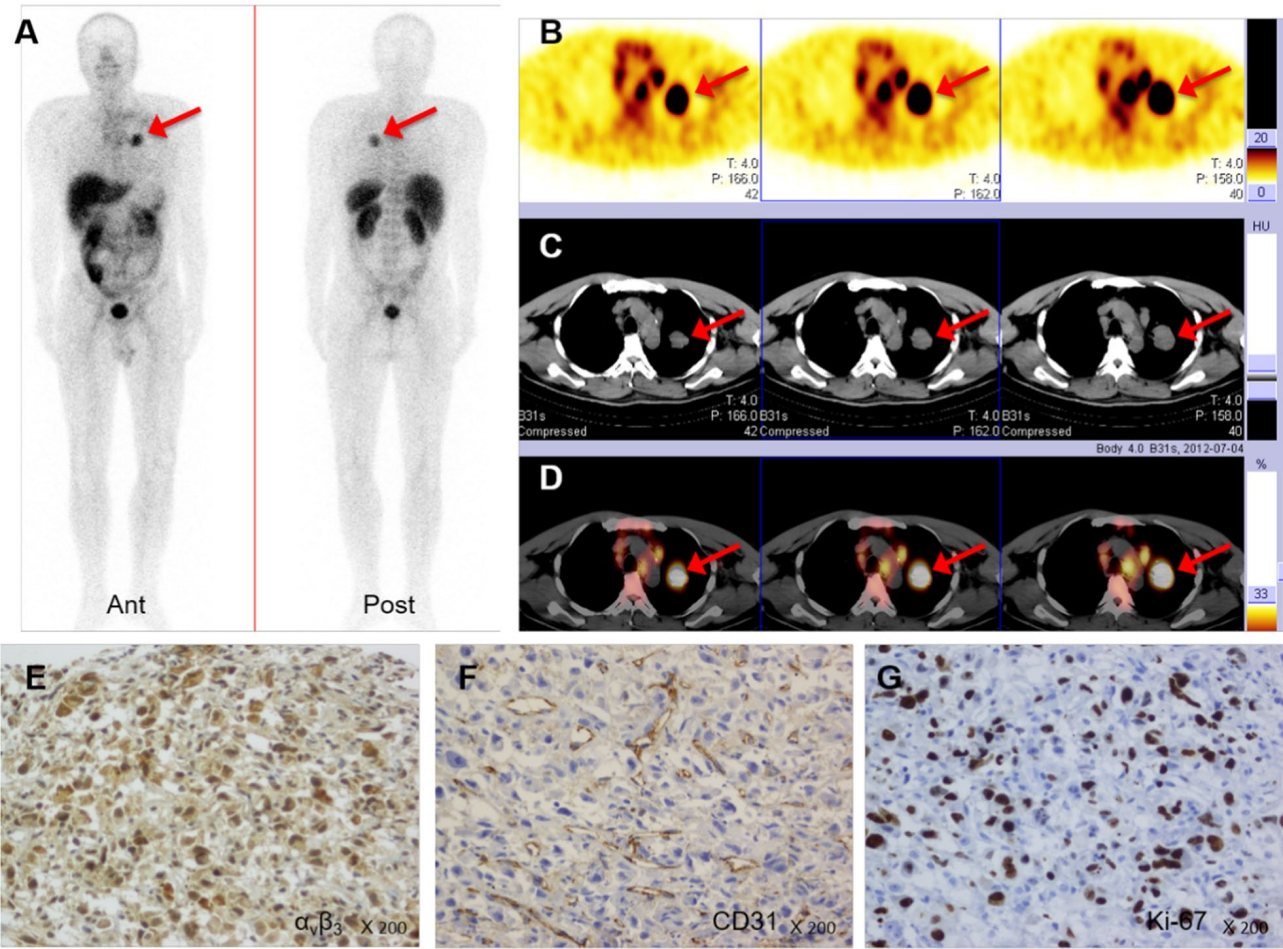
**[**^99m^Tc]Tc-Galacto-RGD_2_ SPECT/CT detected the primary tumour and multiple lymph node metastases in advanced adenocarcinoma. [^99m^Tc]Tc-Galacto-RGD_2_ image (**a**–**d**). **a** Whole-body image. **b**–**d** SPECT/CT. **e**–**g** IHC showed higher expression of α_v_β_3_ in the tumour cells and neo-vasculature, higher density of micro-vessel with CD31 expression in the tumour tissue, and a higher Ki-67 index

**Fig. 7:**
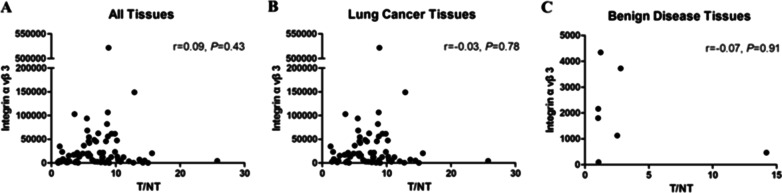
The correlation analysis between ratio of primary tumour to normal tissue (T/NT) and α_v_β_3_ expression in different groups

**Fig. 8:**
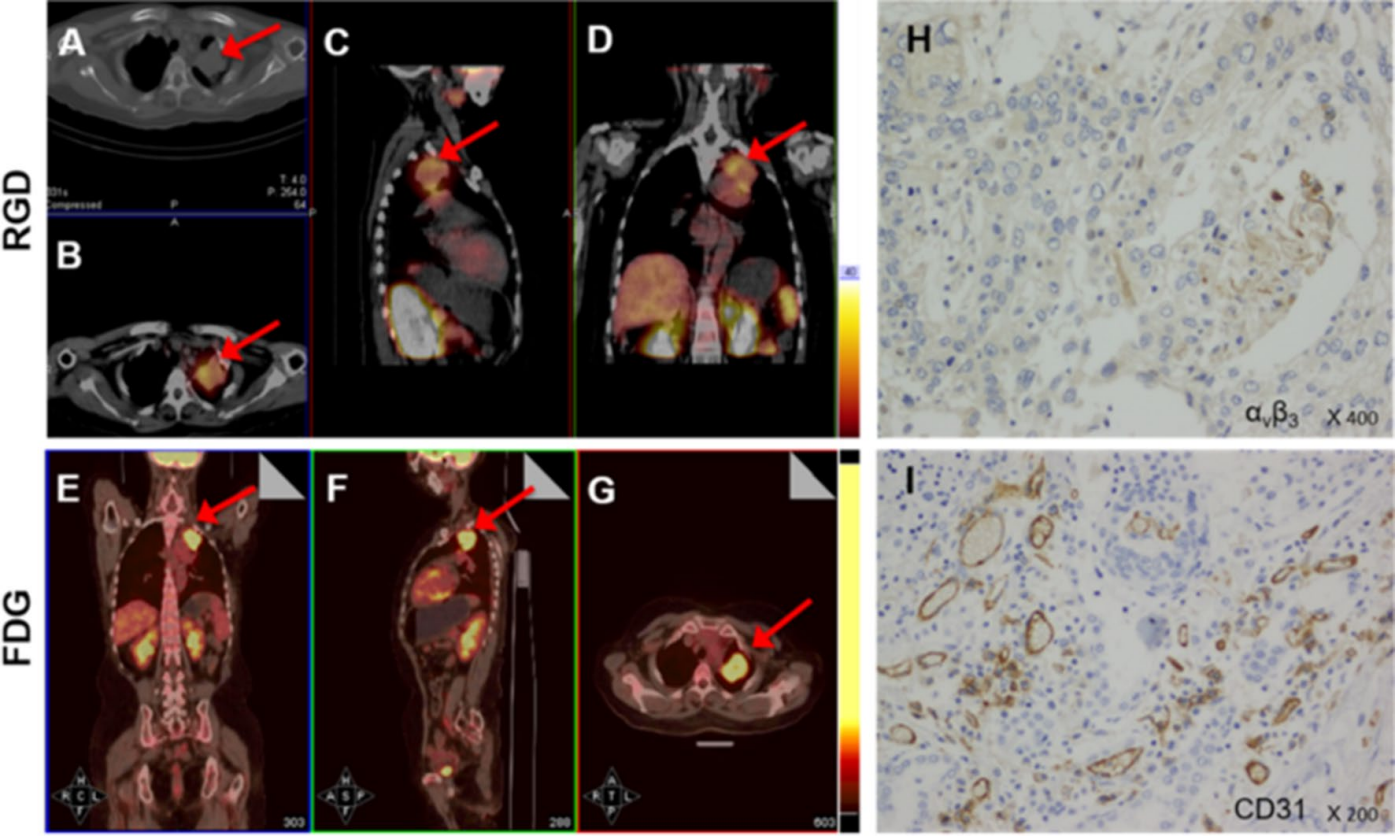
[^99m^Tc]Tc-Galacto-RGD_2_ SPECT/CT showed RGD-avid lesion in a female patient with squamous cell carcinoma. **a**–**d** RGD imaging showed focal uptake in the left lung. **e**–**g** FDG-avid lesion shown in the left upper lung. **h**, **i** IHC showed a moderate α_v_β_3_ expression in the tumour cell membrane, and a high density of neovascularization with high expression of CD31

**Fig. 9:**
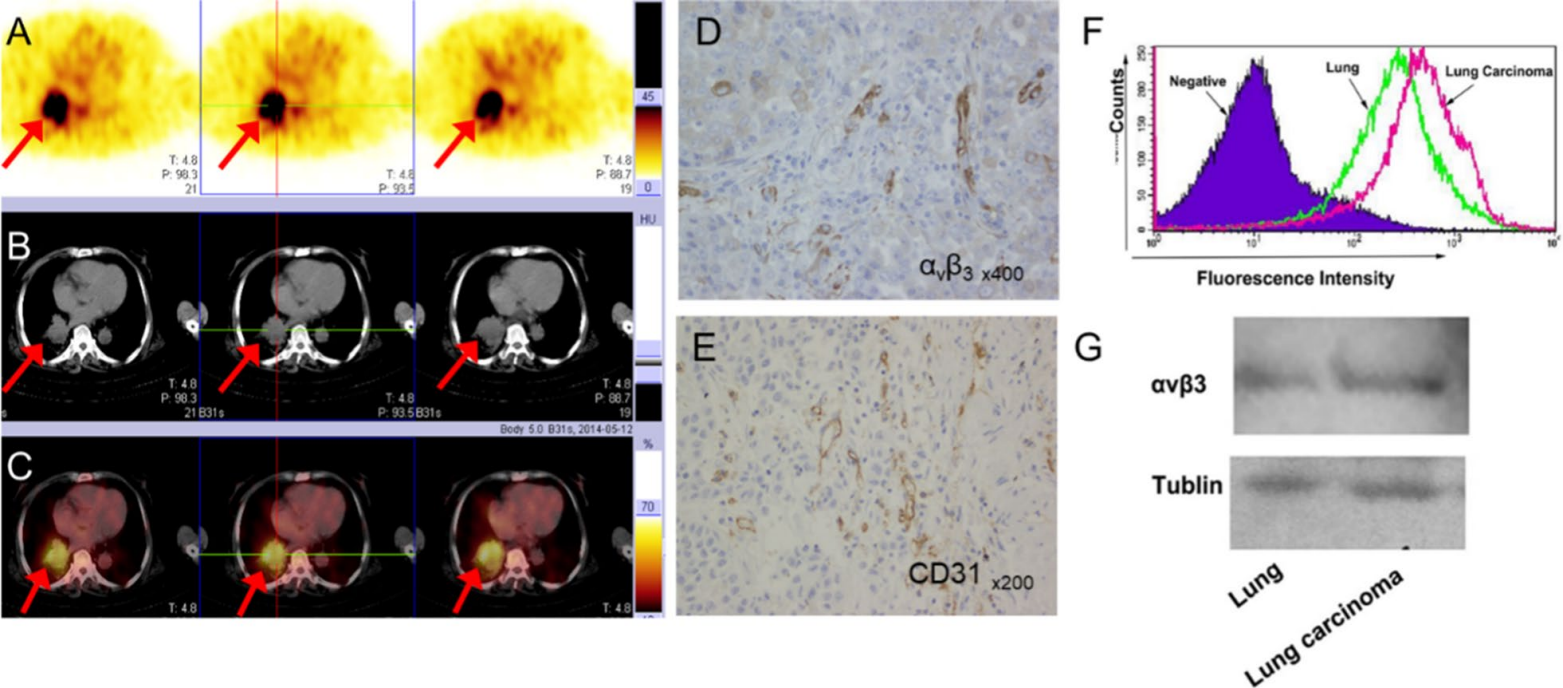
RGD-avid lesion in a patient with lung adenocarcinoma, consistent with α_v_β_3_ expression which confirmed by IHC, flow cytometry and western blot. **a**–**c** [^99m^Tc]Tc-Galacto-RGD_2_ image. **d**, **e** IHC showed higher expression of α_v_β_3_ in the tumour cells and higher density of micro-vessel (CD31). **f** Flow cytometry showed higher expression of integrin β_3_ in tumour tissue compared with normal lung tissue. **g** Western blot showed higher integrin β_3_ expression in primary tumour tissue compared with normal lung tissue

Of the 210 patients with suspected lung cancer, immunohistochemistry (IHC) was performed in 66 patients with lung cancer and seven patients with benign diseases. Expression levels (median (interquartile range)) of integrin α_v_β_3_ were significantly higher in lung cancer (OD: 15,020.5 (4482.6, 44,455.2)) compared with benign diseases (OD: 1797.8 (794.0, 2943.6); *p* < 0.01) (Table 2). CD31 levels were also elevated in lung cancer (OD: 21.9 (13.75, 34.35) vs 9.00 (8.90, 11.50); *p* < 0.01). Higher levels of integrin α_v_β_3_ were expressed in advanced tumours (OD: 19,729.00 (6445.40, 45,288.30)) compared with locoregional tumours (5914.40 (1461.60, 17,658.20)), *p* < 0.05. Integrin α_v_β_3_ was highly expressed not only in endothelial cells in the neovasculature, reflected by CD31 expression, but also in tumour cells (Fig. 6), with a higher density of neovasculature and integrin α_v_β_3_ expression in the primary tumour**.** Integrin α_v_β_3_ was also significantly correlated with CD31 expression in lung cancer (*r* = 0.30, *p* = 0.016). However, there was no correlation between tumour uptake of [^99m^Tc]Tc-Galacto-RGD_2_ and integrin α_v_β_3_ expression in the primary tumour in this study (Fig. 7). Squamous lung cancer usually showed higher level of α_v_β_3_ in the tumour cell and the higher density of microvessel, which was consistent with RGD imaging as shown in Fig. 8. Aggressive LAC tends to higher express integrin α_v_β_3_ in the tumour cell and has denser micro-vessels, which showed focal uptake in the RGD image, as shown in Fig. 9. Neo-vascularization varied in benign respiratory diseases, associated with higher integrin α_v_β_3_ expression. In the current study, integrin α_v_β_3_ correlated with CD31 expression in the neo-vessel, indicating that integrin α_v_β_3_ mediated angiogenesis, leading to tumour development and metastasis. We also examined CXCR4 expression. CXCR4 was highly expressed in lung cancer, as demonstrated by IHC. Furthermore, expression levels of CXCR4 tended to be positively correlated with integrin α_v_β_3_ levels in lung cancer specimens (*r* = 0.22*, p* > 0.05). In addition, the proliferation index (Ki-67, median (interquartile range)) in LSC and SCLC (27.45 (11.88, 42.00) and 70.00 (55.13, 73.48), respectively) were both significantly higher than in LAC (10.15 (2.98, 27.89)) (Table 3).

**Table 2:** Immunohistochemical results of lung cancer and benign disease

Variants	Lung cancer (*n* = 66)	Benign disease (*n* = 7)	*p*
Integrin α_v_β_3_ (OD)	15,020.5 (4482.6,44,455.2)	1797.8 (794.0,2943.6)	**1.08E−03**
CXCR4 (OD)	5120.0 (1978.0,18,460.0)	538.6 (300.0,7101.7)	0.08
CD31 (MVD)	21.9 (13.75,34.35)	9.00 (8.90,11.50)	**5.56E-03**
Ki-67 (%)	20.00 (7.46,40.00)		

Bold values indicate* p* values means there is a significance of the statistical results

*OD* optical density, *MVD* microvessel density. The non-normal distribution data showed as median (interquartile range)

**Table 3:** Immunohistochemical results among different groups of lung cancer

	Stage			Class				T/NT (1 h)		
	I-II (*n* = 21)	III-IV (*n* = 45)	*p*	LAC (*n* = 34)	LSC (*n* = 26)	SCLC (*n* = 6)	*p*	low (< 7.21) (*n* = 33)	high (≥ 7.21) (*n* = 33)	*p*
Integrin α_v_β_3_ (OD)	5914.4 (1461.6, 17,658.2)	19,729.0 (6445.4, 45,288.3)	***3.10E-02***	17,308.1 (7973.6,47,755.9)	9721.6 (3186.5,23,010.7)	6485.0 (3083.0,28,119.0)	0.31	16,958.0 (3842.8,36,479.0)	12,235.4 (4935.2,47,323.5)	0.98
CXCR4 (OD)	8438.0 (2582.5,22,821.8)	4002 (1863,11,541)	0.18	4536 (1348,10,501)	9052.0 (3688.9,33,442.9)	2324 (1980,4395)	0.11	6561.00 (2049.20,18,854.50)	5007 (1335,15,314)	0.65
CD31 (MVD)	28.20 (14.25,34.40)	21.33 (13.75,32.00)	0.54	27.80 (14.44,34.35)	19.72 (15.06,32.11)	13.32 (11.75,20.04)	0.25	25.80 (15.00,35.40)	18.20 (13.75,28.40)	0.25
Ki67 (%)	11.00 (5.00,40.00)	22.82 (8.63,43.95)	0.47	10.15 (2.98,27.89)	27.45 (11.88,42.00)	70.00 (55.13,73.48)	***5.64E-03***	16.13 (4.36,50.00)	21.90 (10.00,38.90)	0.55

Bold values indicate* p* values means there is a significance of the statistical results

*LAC* adenocarcinoma, *LSC* squamous cell carcinoma, *SCLC* small cell lung cancer, *OD* optical density, *MVD* microvessel density. The non-normal distribution data showed as median (interquartile range)

### Lymphadenopathy and distant metastasis

Of the 185 patients with lung cancer, 116 patients had lymphadenopathy, 87 had remote metastasis, 17 had multiple lung tumours including pleural invasion, and 70 patients had bone metastasis. The metastatic lymph nodes and remote metastases showed high focal uptake of [^99m^Tc]Tc-Galacto-RGD_2_. However, although lymphadenopathy was evaluated by imaging follow-up, the final diagnosis was not confirmed, and we were therefore unable to evaluate the diagnostic value of [^99m^Tc]Tc-Galacto-RGD_2_ imaging for lymphadenopathy and remote metastasis in this study.

## Discussion

We previously validated the ability of [^99m^Tc]Tc-Galacto-RGD_2_ to identify iodine-refractory status in patients with thyroid cancer [27]. In a rare case with a solitary fibrous tumour located in the main pulmonary artery, [^99m^Tc]Tc-Galacto-RGD_2_ imaging played an important role in detecting the primary tumour and predicting the metastatic potential [22]. In the current study, we evaluated the use of [^99m^Tc]Tc-Galacto-RGD_2_ SPECT/CT for the detection of lung cancer. We also explored the expression of integrin α_v_β_3_ and CXCR4 in different lung cancer subtypes, and compared the neovasculature among these subtypes. The correlations between tumour uptake of [^99m^Tc]Tc-Galacto-RGD_2_ and integrin α_v_β_3_ expression and neovascularization were also explored.

High-contrast images of [^99m^Tc]Tc-Galacto-RGD2 showed a significantly higher T/NT ratio in malignant compared with benign lung lesions. Malignant primary tumours and metastatic lymph nodes showed higher focal uptake, while benign lesions showed significantly lower uptake. [^99m^Tc]Tc-Galacto-RGD_2_ SPECT/CT showed high sensitivity for primary tumours and remote metastases. ROC analysis showed a sensitivity and accuracy of 91.89% and 86.67%, respectively, for [^99m^Tc]Tc-Galacto-RGD_2_ SPECT/CT, using a cut-off value of T/N ratio at 2.5. However, the specificity for differentiating between malignant and benign disease was limited, possibly because of the involvement of integrin α_v_β_3_ in various benign diseases. Overlap usually occurs between tuberculosis and inflammatory pseudo-tumours, which usually show higher uptake of [^99m^Tc]Tc-Galacto-RGD_2_ than other types of benign diseases, such as pneumonia [12].

In the current study, IHC showed that α_v_β_3_ levels were higher in advanced lung cancer, and proliferation index, represented by Ki-67, was significantly increased in advanced stages of SCLC, associated with metastatic potential [12, 19, 28]. Patients with lung cancer, even in the early stages, may develop multiple metastases several months even after thorough tumour resection, possibly related to specific tumour types with higher metastatic potential [19]. In the current study, CXCR4 expression levels were higher in lung cancer compared with benign disease, though the differences were not significant. Its expression was correlated with both integrin α_v_β_3_ and CD31 expression in primary lung tumours, while integrin α_v_β_3_ was also correlated with CD31. These findings validate our hypothesis that lymphadenopathy and remote metastasis are mediated by specific biological molecules. Integrin α_v_β_3_ and CXCR4 may mediate angiogenesis, which may further promote lymph node and remote metastases. Integrin α_v_β_3_-targeted imaging thus improves our understanding of the interactions between cancer cells and their microenvironment, which is a necessary prerequisite for the development of treatment strategies. This study showed higher levels of integrin α_v_β_3_ were expressed in advanced tumours, integrin α_v_β_3_ was also highly expressed not only in endothelial cells in the neo-vasculature but also in tumour cells, higher uptake was found in the primary tumour with a higher density of neo-vasculature and higher α_v_β_3_ expression, which associated with multiple lymphadenopathy and remote metastasis**.** This finding confirmed integrin α_v_β_3_ overexpression as an important component of tumour microenvironment, which was related with tumourigenic and aggressive behaviour in lung cancer. CXCR4 has been implicated in the chemotactic migration of cancer cells [10]. CXCR4 and integrin might synergistically promote lymphatic metastasis in lung cancer, and act as clinical predictors of lymph node metastasis in non-SCLC [29, 30]. High expression levels of chemokines are related to a poor prognosis and a poor chemotherapy tolerance in cancer patients [31–34]. CXCR4 is a chemokine receptor that plays a critical role in the process of lymphocyte homing to lymphatic vessels and secondary lymphoid organs, including the lymph nodes [35].

Integrin α_v_β_3_ was expressed not only in the tumour cells, but also in the endothelium, though there was a lack of a correlation between tumour uptake of **[**^99m^Tc]Tc-Galacto-RGD_2_ and integrin α_v_β_3_ expression related to the heterogenicity of lung cancer. We found that tumour uptake of [^99m^Tc]Tc-Galacto-RGD_2_ was related to integrin α_v_β_3_ expression, neovascularization, and tumour stage, and integrin α_v_β_3_ expression in tumour cells may promote lymphatic and distant metastases (Fig. 2**)**. However, benign diseases showed variable degrees of angiogenesis, also associated with higher expression of integrin α_v_β_3_, as shown in one patient with thymus adenoma and in another with pulmonary sequestration (Figs. 3, 4). We hypothesized that tumour uptake of [^99m^Tc]Tc-Galacto-RGD_2_ depended on the neo-vasculature and integrin α_v_β_3_ expression in the tumour cells, and focal uptake in RGD-targeted imaging would thus be higher in primary tumours with more neo-vasculature and higher integrin α_v_β_3_ expression in the cell membrane. Regarding the different subtypes of lung cancer, LSC had more neovascularization and higher integrin α_v_β_3_ expression, followed by LAC, while SCLC showed less neovascularization and a higher proliferation index. The highest T/N ratio was therefore found in LSC, and was significantly higher than that in LAC and SCLCs. RGD-targeted imaging may thus serve as a useful tool for the phenotyping of lung cancer.

[^68^ Ga]Ga and [^18^F]F labelled RGD tracers have been used in the preclinical and clinical, [^68^ Ga]Ga-NODAGA-RGD provide a different spatial distribution than 2-[^18^F]FDG. It is worth noting that [^18^F]F-Galacto-RGD not only can be used for the assessment of α_v_β_3_ expression in the tumour neovasculature, but also in human atherosclerotic carotid plaques, where it correlates with α_v_β_3_ expression [36]. Compared with PET RGD tracer, [^99m^Tc]Tc-Galacto-RGD_2_ SPECT imaging has a disadvantage in space resolution. However, if taking the expenditure into account, [^99m^Tc]Tc-Galacto-RGD_2_ has a significant advantage, and the one-kit vial of [^99m^Tc]Tc-Galacto-RGD_2_ makes the synthesis more convenient, both of them contribute to the clinical transformation and application of [^99m^Tc]Tc-Galacto-RGD_2_.

However, there are some limitations in this study should be taken into concern. First, the quantitation of integrin α_v_β_3_ expression in the immunohistochemistry could be influenced by tumour specimen obtaining and vision field selection. Second, tumour specimens were achieved only in 66 patients, not in all suspected patients, which might influence the data analysis.

## Conclusions

This was the first extensive longitudinal study to investigate the expression of integrin α_v_β_3_ in lung cancer. [^99m^Tc]Tc-Galacto-RGD_2_ imaging showed high sensitivity for the detection of primary lung cancer, but limited specificity. [^99m^Tc]Tc-Galacto-RGD_2_ uptake in the primary tumour was attributed to integrin α_v_β_3_ expression in the endothelial and tumour cells, and focal uptake occurred in primary lung cancers with more neovascularization and high levels of α_v_β_3_ in the tumour cells. LSC had a higher density of neo-vessels and higher α_v_β_3_ expression, followed by LAC and then SCLC, advanced lung cancer showed higher levels of integrin α_v_β_3_ compared with early stage. These findings suggest that RGD-based imaging might be a useful tool for lung cancer phenotyping and tumour biological behaviour evaluation. Further studies are warranted to validate these findings.

## Data Availability

The datasets used and analysed during the current study are available from the corresponding author upon reasonable request.

## Abbreviations

TKIs: Tyrosine kinase inhibitors
SPECT/CT: Single-photon emission computed tomography
T/N: Tumour/normal tissue ratio
SCLC: Small cell lung cancer
LSC: Squamous cell carcinoma
LAC: Adenocarcinoma
EGFR-TKIs: Epidermal growth factor receptor-tyrosine kinase inhibitors
ASCO: The American Society of Clinical Oncology
PET/CT: Positron emission tomography/computed tomography
RGD: Arginine-glycine-aspartate
ROI: Region of interest
H&E: Haematoxylin and eosin
OD: Optical density
MVD: Microvessel density
AUC: Area under the curve
ROC: Receiver operating characteristic
IHC: Immunohistochemistry

## References

[1] Bray F, Ferlay J, Soerjomataram I, Siegel RL, Torre LA, Jemal A (2018). Global cancer statistics 2018: GLOBOCAN estimates of incidence and mortality worldwide for 36 cancers in 185 countries. CA Cancer J Clin.

[2] Chen W, Zheng R, Baade PD, Zhang S, Zeng H, Bray F (2016). Cancer statistics in China, 2015. CA Cancer J Clin.

[3] Yang D, Liu Y, Bai C, Wang X, Powell CA (2020). Epidemiology of lung cancer and lung cancer screening programs in China and the United States. Cancer Lett.

[4] Zhang H, Cai B (2003). The impact of tobacco on lung health in China. Respirology.

[5] Arbour KC, Riely GJ (2019). Systemic therapy for locally advanced and metastatic non-small cell lung cancer: a review. JAMA.

[6] Travis WD, Brambilla E, Nicholson AG, Yatabe Y, Austin JHM, Beasley MB (2015). The 2015 world health organization classification of lung tumors: impact of genetic, clinical and radiologic advances since the 2004 classification. J Thorac Oncol.

[7] Ferguson FM, Gray NS (2018). Kinase inhibitors: the road ahead. Nat Rev Drug Discov.

[8] Baeriswyl V, Christofori G (2009). The angiogenic switch in carcinogenesis. Semin Cancer Biol.

[9] Zaidel-Bar R (2013). Job-splitting among integrins. Nat Cell Biol.

[10] Wu D, Xu Y, Ding T, Zu Y, Yang C, Yu L (2017). Pairing of integrins with ECM proteins determines migrasome formation. Cell Res.

[11] Ruoslahti E, Pierschbacher MD (1987). New perspectives in cell adhesion: RGD and integrins. Science.

[12] Niu G, Chen X (2011). Why integrin as a primary target for imaging and therapy. Theranostics.

[13] Demircioglu F, Hodivala-Dilke K (2016). alphavbeta3 Integrin and tumour blood vessels-learning from the past to shape the future. Curr Opin Cell Biol.

[14] Sun X, Ma T, Liu H, Yu X, Wu Y, Shi J (2014). Longitudinal monitoring of tumor antiangiogenic therapy with near-infrared fluorophore-labeled agents targeted to integrin alphavbeta3 and vascular endothelial growth factor. Eur J Nucl Med Mol Imaging.

[15] Joseph JM, Gross N, Lassau N, Rouffiac V, Opolon P, Laudani L (2005). In vivo echographic evidence of tumoral vascularization and microenvironment interactions in metastatic orthotopic human neuroblastoma xenografts. Int J Cancer.

[16] Zou W, Teitelbaum SL (2015). Absence of Dap12 and the alphavbeta3 integrin causes severe osteopetrosis. J Cell Biol.

[17] Zhang L, Meng X, Shan X, Gu T, Zhang J, Feng S (2019). Integrin alphavbeta3-specific hydrocyanine for cooperative targeting of glioblastoma with high sensitivity and specificity. Anal Chem.

[18] Wang T, Li G, Wang D, Li F, Men D, Hu T (2019). Quantitative profiling of integrin alphavbeta3 on single cells with quantum dot labeling to reveal the phenotypic heterogeneity of glioblastoma. Nanoscale.

[19] Wu FH, Luo LQ, Liu Y, Zhan QX, Luo C, Luo J (2014). Cyclin D1b splice variant promotes alphavbeta3-mediated adhesion and invasive migration of breast cancer cells. Cancer Lett.

[20] Krishn SR, Singh A, Bowler N, Duffy AN, Friedman A, Fedele C (2019). Prostate cancer sheds the alphavbeta3 integrin in vivo through exosomes. Matrix Biol.

[21] Yan B, Qiu F, Ren L, Dai H, Fang W, Zhu H (2015). (99m)Tc-3P-RGD2 molecular imaging targeting integrin alphavbeta3 in head and neck squamous cancer xenograft. J Radioanal Nucl Chem.

[22] Luo R, Jiang Y, Huang Y, Chen X, Wang F (2020). Longitudinal observation of solitary fibrous tumor translation into malignant pulmonary artery intimal sarcoma. J Cardiothorac Surg.

[23] Yan B, Fu T, Liu Y, Wei W, Dai H, Fang W (2018). 99mTc-3PRGD2 single-photon emission computed tomography/computed tomography for the diagnosis of choroidal melanoma: a preliminary STROBE-compliant observational study. Medicine (Baltimore).

[24] Fu T, Qu W, Qiu F, Li Y, Shao G, Tian W (2014). (99m)Tc-3P-RGD2 micro-single-photon emission computed tomography/computed tomography provides a rational basis for integrin alphavbeta3-targeted therapy. Cancer Biother Radiopharm.

[25] Ji S, Czerwinski A, Zhou Y, Shao G, Valenzuela F, Sowinski P (2013). (99m)Tc-Galacto-RGD2: a novel 99mTc-labeled cyclic RGD peptide dimer useful for tumor imaging. Mol Pharm.

[26] Kay FU, Kandathil A, Batra K, Saboo SS, Abbara S, Rajiah P (2017). Revisions to the tumor, node, metastasis staging of lung cancer (8 (th) edition): rationale, radiologic findings and clinical implications. World J Radiol.

[27] Xu Q, Liu R, Wang J, Huang Y, Li S, Zhang L (2020). Role of [(99m)Tc]Tc-Galacto-RGD2 SPECT/CT in identifying metastatic differentiated thyroid carcinoma after thyroidectomy and radioactive iodine therapy. Nucl Med Biol.

[28] Hoster E, Rosenwald A, Berger F, Bernd HW, Hartmann S, Loddenkemper C (2016). Prognostic value of Ki-67 index, cytology, and growth pattern in mantle-cell lymphoma: results from randomized trials of the european mantle cell lymphoma network. J Clin Oncol.

[29] Chen FH, Fu SY, Yang YC, Wang CC, Chiang CS, Hong JH (2013). Combination of vessel-targeting agents and fractionated radiation therapy: the role of the SDF-1/CXCR4 pathway. Int J Radiat Oncol Biol Phys.

[30] Iwakiri S, Mino N, Takahashi T, Sonobe M, Nagai S, Okubo K (2009). Higher expression of chemokine receptor CXCR7 is linked to early and metastatic recurrence in pathological stage I nonsmall cell lung cancer. Cancer.

[31] Demir IE, Mota RC (2020). Chemokines: the (un)usual suspects in pancreatic cancer neural invasion. Nat Rev Gastroenterol Hepatol.

[32] Peng D, Kryczek I, Nagarsheth N, Zhao L, Wei S, Wang W (2015). Epigenetic silencing of TH1-type chemokines shapes tumour immunity and immunotherapy. Nature.

[33] Lau S, Feitzinger A, Venkiteswaran G, Wang J, Lewellis SW, Koplinski CA (2020). A negative-feedback loop maintains optimal chemokine concentrations for directional cell migration. Nat Cell Biol.

[34] Kwon D, Lozada J, Zhang Z, Zeisler J, Poon R, Zhang C (2021). High-contrast CXCR4-targeted (18)F-PET imaging using a potent and selective antagonist. Mol Pharm.

[35] Cabioglu N, Yazici MS, Arun B, Broglio KR, Hortobagyi GN, Price JE (2005). CCR7 and CXCR4 as novel biomarkers predicting axillary lymph node metastasis in T1 breast cancer. Clin Cancer Res.

[36] Beer AJ, Pelisek J, Heider P, Saraste A, Reeps C, Metz S (2014). PET/CT imaging of integrin alphavbeta3 expression in human carotid atherosclerosis. JACC Cardiovasc Imaging.

